# The new insight of treatment in Cholangiocarcinoma

**DOI:** 10.7150/jca.68264

**Published:** 2022-01-01

**Authors:** Yuhang Li, Yinghui Song, Sulai Liu

**Affiliations:** 1 Department of Hepatobiliary Surgery, Hunan Provincial People's Hospital/The First Affiliated Hospital of Hunan Normal University, Changsha, 410005 Hunan Province, China.; 2 Hunan Research Center of Biliary Disease, Changsha, 410005 Hunan Province, China.; 3 Central Laboratory of Hunan Provincial People's Hospital/The First Affiliated Hospital of Hunan Normal University, Changsha, 410015, China.

**Keywords:** Cholangiocarcinoma, Insight, Treatment

## Abstract

Cholangiocarcinoma (CCA) is a relatively rare malignant tumor originating from the bile duct epithelial cells, and it is one of the malignant tumors with fast growth in incidence and death rate in recent years. CCA carries a very poor prognosis due to a typically late clinical presentation and a poor response to current therapeutics. Currently, surgery is the only possible curative treatment, radiotherapy and chemotherapy also play an important role in slowing down disease progression, while targeted therapy and immunotherapy are changing with each passing day and their combined effect may have great potential for the treatment of CCA; Clinical trials of various treatment options for CCA are also being conducted. This article reviews the different treatment options for CCA and explores the adjuvant treatment for it from a new perspective. In the future, the goal of treatment should be multiple and combined for different CCA patients to achieve individualized programs and improve overall survival.

## Introduction

Cholangiocarcinoma is an epithelial cell malignancy arising at different locations within the biliary tree showing marks of cholangiocyte differentiation 1, 2. They are usually classified by anatomical location into intrahepatic cholangiocarcinoma (ICCA) 3, perihilar cholangiocarcinoma (PCCA) and distal cholangiocarcinoma (DCCA), the latter two often collectively referred to as extrahepatic cholangiocarcinoma (ECCA), each has a separate American Joint Committee on Cancer (AJCC) staging system 4. ICCA is defined as bile duct cancer located at the proximal end of the secondary bile duct (proximal and distal refers to the direction of bile flow so that the intrahepatic bile duct is close to the common bile duct); In the liver, PCCA is located in the area of the secondary bile duct where the cystic duct is inserted into the common bile duct; and DCCA is limited to the area between the origin of the cystic duct and the ampulla of Vater 5. The overall incidence of CCA has been increasing globally over the past 40 years 6. The absence of specific clinical manifestations in the early stages of the disease makes diagnosis difficult, and most patients do not develop significant clinical symptoms until the disease has progressed to intermediate or advanced stages, but treatment options are very limited at this time and the prognosis is poor 7. In recent years, the treatment methods for CCA have become more and more diversified, including more mature and effective surgical treatment, radiotherapy and chemotherapy in the first-line treatment, especially immunotherapy, targeted therapy and combination therapy that have recently become hot spots. However, there has not been a good consensus on the treatment of CCA, and the development of individualized treatment and comprehensive treatment plans for different patients has not been well recognized and popularized. Based on this, this article reviews the treatment of CCA, discusses the different therapeutic approaches or combined treatment options for CCA, and it systematically introduces the effects of different treatments for CCA and the clinical benefits of patients.

## Surgery

**Figure 1:**
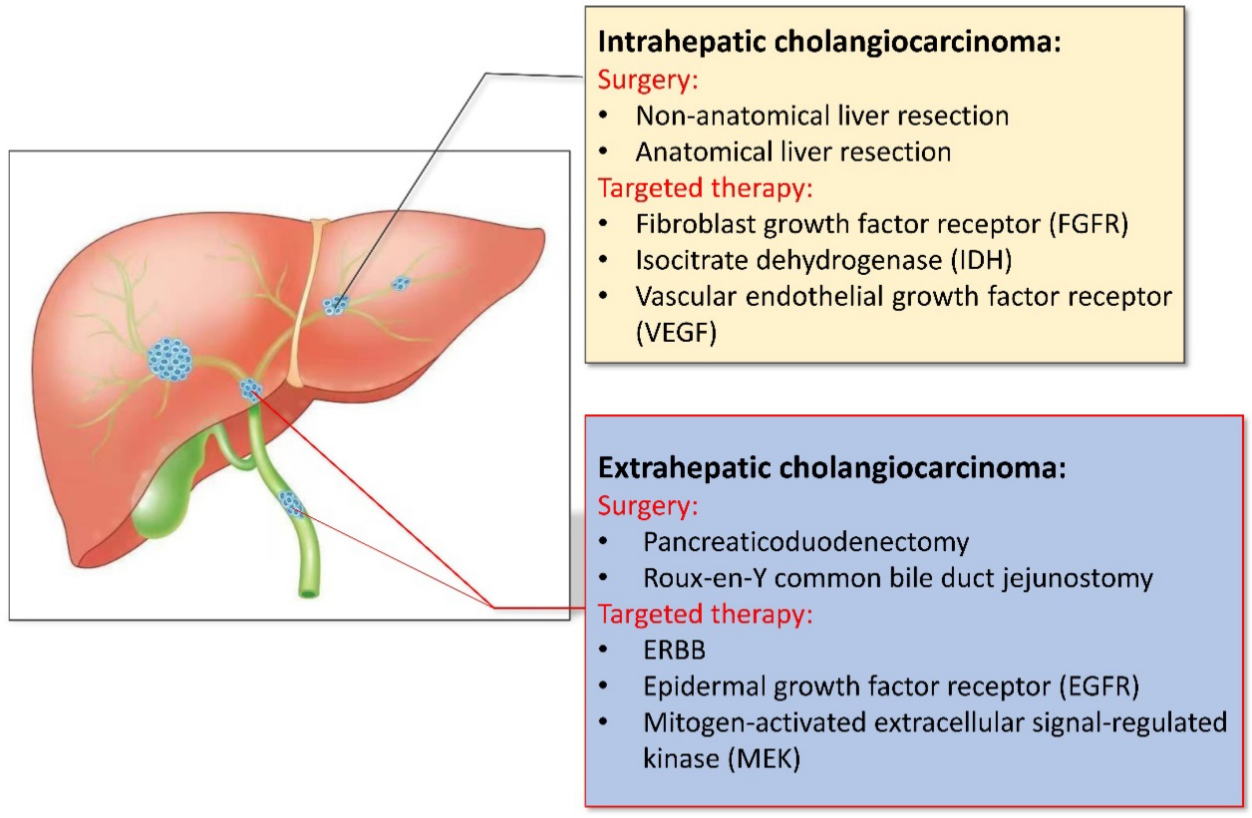
Different treatment of intrahepatic and extrahepatic cholangiocarcinoma.

The early symptoms of CCA are not obvious, studies have shown that only about 35% of patients can be detected early and receive surgical treatment, and most of the patients have already developed advanced stages by the time of presentation. For patients who may undergo surgical treatment, we need to think about how to safely perform surgical treatments 8. Accurate preoperative evaluation is very necessary for patients, not only can it effectively increase the success rate of surgery, but also improves the quality of life and prognosis of patients. When the patient has the conditions for surgery, it is necessary to consider the method of surgery, the scope of resection, and the need for lymph node dissection. In view of the differences in surgical methods and prognosis of different types of CCA, which will be analyzed separately (shown in Fig. 1).

For ICCA, radical surgical treatment is considered the only truly effective treatment, based on the principle of margin-negative hepatectomy with preservation of a postoperative future remnant liver (FLR) of adequate size and function. The median overall survival (OS) after radical resection is about 30 months, and the 5-year overall survival rate after surgery is generally up to 40%, but patients with negative margins (R0 resection) and lymph node involvement can get better survival rates. The 5-year survival rate can be as high as 63% 9. Many centers have recommended an aggressive surgical approach involving extensive hepatectomy with expanded systemic lymph node dissection to improve prognosis 10. Patients should undergo surgery only if they have potentially resectable tumors and are suitable candidates for surgery 11. In contrast, there was no significant difference in perioperative complications and mortality in patients treated with anatomic resection (AR) of the liver compared to those treated with non-anatomic resection (NAR) of the liver. Patients treated with AR can have a better long-term prognosis, especially for ICCA patients with TNM stage I tumors >5 cm and TNM stage II without vascular invasion 12. In 2015, an international consensus based on the available data at the time concluded that lymphadenectomy may improve the staging and prognosis. It is recommended that patients with lymph node metastasis (LNM) undergo standard hepatoduodenal lymph node dissection in the surgical treatment of ICCA 13. However, Zhang XF et al. did a retrospective study and confirmed that the prediction model for predicting LNM before surgery was not effective based on existing data. Routine histological evaluation of lymph node resection seems to be the only accurate method for diagnosing LNM and providing accurate staging. Therefore, the results of lymph node dissection can be used to guide staging and determine the indications for adjuvant chemotherapy 14. Several other studies have shown that LNM has no effect on the survival of patients without evidence of LNM 15. At the same time, preventive lymph node dissection should be performed with caution in patients with liver cirrhosis, because it is associated with a significant increase in postoperative complications 16. Therefore, LN anatomy should be limited, and individualizing plans should be developed for different patients, preferably only for obtaining samples for staging and other purposes. Liver transplantation is also an alternative surgical treatment. Although ICCA is considered a contraindication for liver transplantation, studies have shown that for early ICCA smaller than 2 cm, liver transplantation can provide patients with better curative effect and survival, and their 1, 3, and 5-year survival rates can reach respectively 93%, 84% and 65% 17. And the results published in 2016 support liver transplantation as a treatment option for early ICCA patients. Compared with other treatments, liver transplantation is more effective. A recent study in Japan also showed that the 5-year survival rate of patients with early ICCA was 82% 18.

Surgical resection of PCCA is a potential treatment option for patients with PCCA without the following exclusion barriers: bilateral secondary bile duct involvement, bilateral or contralateral vascular involvement, presence of metastatic disease, and underlying primary sclerosing cholangitis (PSC) 19. The basic principle of surgery is marginal negative complete resection of the tumor and regional lymph node dissection with the need for resection and reconstruction of the portal vein and/or hepatic artery, and with biliary reconstruction usually performed by Roux-en-Y choledojejunostomy. For some patients with early stages cannot rule out surgical contraindications, the tumor can be removed. Expanding the scope of resection, including three-segment resection, vascular resection and hepatopancreatoduodenectomy, can avoid unnecessary liver transplantation of PCCA, and even provide good results in some truly localized PCCA patients 20. Chen KJ et al. collected the clinical results of 176 patients with advanced PCCA. In the entire cohort, the 1-year, 2-year, and 3-year OS rates were 53%, 24%, and 13%, respectively. According to the treatment method, the OS rates at 1, 2, and 3 years were 65%, 38%, and 38% in the radical resection group (n=62), and 63%, 30%, and 20% in the palliative resection group (n= 28), it can be inferred that surgery is still the best treatment for advanced PCCA 21. Most reported 3-year and 5-year survival rates after PCCA resection are approximately 45% (35% to 60%) and 30% (15% to 40%) 22. Futural research should focus on improving the R0 resection rate. It can be achieved by more extensive resection, especially the resection of the proximal bile duct. The selection criteria for candidates who are suitable for liver transplantation include: the presence of unresectable tumors with a radial diameter of <3 cm, and the absence of intrahepatic or extrahepatic metastatic disease 23. In the case of PSC, regardless of resectability, the best treatment for PCCA is liver transplantation 22, because visual field defects associated with this underlying chronic liver disease can promote canceration. Therefore, for early patients, the benefits of choosing liver transplantation still need to be verified by more clinical results.

DCCA is associated with the proliferation of connective tissue and the infiltration of surrounding structures, and a preliminary preoperative evaluation is required to rule out distant metastases and assess local resectability. For resectable tumors and patients who can tolerate radical surgery, the surgical option is usually pancreaticoduodenectomy with biliary tract reconstruction 24. In a Mayo Clinic cohort of patients, the median OS of DCCA was 22.0 months 25. In V. Sallinen's study, the OS of DCCA patients was 40.4 months (95% CI: 19.1-61.7 months) 26. In the study of Zhou WW et al., the median OS of all patients was 45.5 months (95% CI 13.5-77.5 months), and the 1-year, 3-year, and 5-year OS rates after resection were 82.6%, 55.2%, and 47.2%, respectively 27.

## Chemotherapy

Chemotherapy plays a certain role in delaying the progression of the disease for patients who are inoperable, even some inoperable patients can receive surgical treatment after induction treatment to obtain higher therapeutic effects. At the same time, chemotherapy is also one of the adjuvant treatments after surgery. Nowadays, with the development of more clinical trials of combination drugs, more appropriate and effective treatment plans can be formulated according to the efficacy of different chemotherapeutic drugs. According to the 2021 NCCN guidelines, regardless of anatomic disease subtype, and the tumor is or not applicable to local and surgical options, the combination of Gemcitabine and Cisplatin remains the current first-line chemotherapy regimen for patients with advanced CCA. Therefore, in view of the fact that the difference in chemotherapy of different types of CCA is not very large, we will discuss them together next. Previously, the Japanese JCOG1113 (FUGA-BT) 28 compared Gemcitabine combined with TGO and Gemcitabine combined with Cisplatin, which showed that Gemcitabine combined with TGO was no less than Gemcitabine combined with cisplatin (OS: 13.4 and 15.1 months, respectively, P=0.06; PFS: 5.8 and 6.8 months, respectively), coupled with the convenience of not hydrating Gemcitabine combined with TGO, it can be considered as a new standard first-line chemotherapy for advanced CCA 29. In addition, the Kansai Hepatobiliary Oncology Group (KHBO1401-MIT-SUBA study; NCT02182778) conducted a phase III trial to evaluate Gemcitabine + Cisplatin + S-1 combination therapy (GCS) over GC therapy in terms of MST. Yanagimoto, H et al. proved that GCS therapy is better than GC 30. Research on Capecitabine, with results from three phase III clinical trials (BCAT, PRODIGE-12/ACCORD-18, BILCAP) 31-33, of which the BILCAP trial showed the presence of adjuvant therapy BILCAP included 447 postoperative patients, randomized to the single-agent Capecitabine group (223 patients) and the observation group (224 patients), the purpose of the trial is to determine the OS difference of the intention-to-treat (ITT) population based on factors such as anatomical location, resection margin status, lymphatic status and many more. The results of the trial showed that the median OS in the ITT population was 51.1 months (95% CI 34.6-59.1 months) and 36.4 months (95% CI 29.7-44.5 months) in the Capecitabine group and the observation group, respectively. The recurrence free survival (RFS) was 24.4 months (95% CI 18.6 to 35.9 months) and 17.5 months (95% CI 12.0 to 23.8 months) in the two groups, with HR 0.75 (95% CI 0.58 to 0.98), p=0.033; based on the data, we can clearly see that the Capecitabine group showed a survival benefit and was well tolerated, thus favoring the prolonged survival of patients. In addition, phase II clinical trials of albumin-conjugated paclitaxel combined with Gemcitabine were also conducted in patients with advanced CCA and achieved good results 34. The combination of albumin-conjugated paclitaxel with chemotherapeutic agents for the first-line treatment of patients with advanced CCA has a survival benefit that is not inferior to that of standard therapy and is tolerable in terms of toxicity, which can be an alternative choice in the clinic. At present, until the advanced CCA -06 study, presented at the 2019 Annual Meeting of American Oncology, establishes oxaliplatin combined with 5-fluorouracil and folic acid as the standard regimen after some treatment failures, there is no standard second-line treatment regimen 35. For patients after surgical resection, a recent trial included 931 adults (18 to 83 years old) who underwent radical resection of CCA. The final result cannot determine the effect of postoperative adjuvant chemotherapy and no postoperative adjuvant chemotherapy on the mortality of patients 36. The role of adjuvant therapy after R0 resection for patients with intrahepatic and perihilar cholangiocarcinoma, for some patients who cannot be operated on, induction therapy can be considered first. Ali Belkouz et al. did a cohort of 10 studies that represented 334 patients with locally advanced PCCA and ICCA who received induction therapy. 180 patients (53.9%) underwent resection after induction therapy, 115 patients (63.9%) underwent R0 resection. Combined OS data showed that chemotherapy plus resection was better than chemotherapy alone (HR = 0.31, 95% CI = 0.19-0.50; P <0.0001). In addition, in studies included, the treatment was well tolerated and the incidence of toxicity was low. These findings may indicate that initially locally advanced PCCA or ICCA is safe and feasible for patients selected for surgery after induction therapy 37. In the later stage, we can continue to study the clinical effects of better chemotherapy drugs. In the future, the best adjuvant chemotherapy treatment for patients with cholangiocarcinoma can be explored 38, and the prognosis of the patient can be improved and the quality of life can be improved.

## Radiation therapy

In the NCCN guidelines, for patients with CCA with positive resection margins or regional LNM under the microscope or naked eye, radiotherapy is recommended after surgery, and this treatment is also considered to be locally advanced unresectable CCA an effective local treatment method. The SEER 39 analysis of 3,839 cases of primary ICCA showed that the median OS of surgery plus adjuvant radiotherapy was 11 months compared with 6 months of surgery alone (p=0.014). Adjuvant radiotherapy after surgery had the greatest benefit on OS (HR=0.40). Gerhards reported a median survival of 24 months for HCC patients who received adjuvant radiotherapy compared to 8 months for surgery alone. Due to the lack of randomized trials, 10 retrospective cohort studies 40 were included in a systematic review and meta-analysis of adjuvant radiation therapy for patients with primary liver cancer. The results showed improved OS with adjuvant three-dimensional conformal radiation therapy (HR=0.62, 95% CI: 0.48-0.78, p<0.001). Stereotactic body radiotherapy (SBRT) is a highly precise radiotherapy technology that focuses on the radical high dose of radiotherapy to the tumor site through external irradiation to achieve the goal of eradicating the tumor. SBRT combined with chemotherapy has shown some efficacy in unresectable biliary tract malignancies and may be a valuable clinical treatment option 41. And Mahadevan A et al. 42 did an analysis of the efficacy of SBRT for CCA and showed that SBRT not only improved the overall survival of patients, but also its radiation toxicity was less, and its radiotoxicity is mild. In several prospective and retrospective studies 43, SBRT resulted in local control rates ranging from 65-100% in selected patients with a median OS of 11-35.5 months (15 months). In a systematic review including 10 studies and 231 patients 32, the aggregated 1-year LC was 83.4% (95% CI: 76.5-89.4%). Meanwhile, the combination of stenting with palliative external radiotherapy and/or brachytherapy may improve stent patency and survival. In addition, the studies on unresectable ICCA and particle radiotherapy are all phase I/II trials or small sample retrospective studies. However, regardless of tumor size, there is a good local control rate and long-term survival is also achieved 44. Newer advanced radiation technologies offer scope for achieving better disease control and reduced morbidity 45. Leng KM et al. conducted a study of 1917 patients with PCCA, the OS of patients receiving adjuvant radiotherapy and surgery was only 23 months and 22 months (P=0.651). In the matched population, radiation therapy did not show better OS or cancer-specific survival (CSS) (17 months vs 18 months, P=0.554). Therefore, it is inferred that there is no obvious relationship between radiotherapy and the improvement of survival rate of patients with PCCA resection 46. And a Phase I feasibility study in the STRONG trial about SBRT for unresectable PCCA, fractionated SBRT after standard chemotherapy is a viable and safe local treatment option for unresectable PCCA 47. A recent data analysis on DCCA found that adjuvant radiotherapy after DCCA resection is related to the survival benefits of patients, even in patients with negative resection margins or lymph node resections. Regardless of the resection margin and lymph node status after DCCA resection, adjuvant radiotherapy should be considered routinely 33, 48. Due to the high local recurrence rate and low survival rate after radical surgery, postoperative radiation therapy plays an important role and effective combination therapy combined with radiation therapy is the direction to improve the survival rate of patients with CCA. Nowadays, although there is no strong evidence that radiotherapy has significant benefits for some types of patients, with the increase in sample size and the deepening of research, we can try radiotherapy or combination therapy for some inoperable or postoperative patients in the future.

## Targeted therapy

Targeted therapy is a treatment method that targets the identified carcinogenic sites at the cellular and molecular level. Corresponding therapeutic drugs can be designed, when the drug enters the body, it will specifically select the carcinogenic site to combine and act, causing the specific death of tumor cells. Without affecting the normal tissue cells surrounding the tumor. With the development of next-generation gene sequencing technology and the discovery of some emerging targets, targeted therapy is becoming a hot topic in the treatment of CCA. A study from the 2020 American Society of Clinical Oncology Gastrointestinal Oncology Symposium (ASCO GI), testing was performed on 212 patients with biliary tract tumors, and 68 patients with drug-available targets were screened for targeted therapy. The median progression-free survival (mPFS) was 6.2 months and 2.8 months, compared to patients who did not receive targeted therapy, with significant clinical outcomes 49. Next, it can be discussed separately according to different sites of action (shown in Fig. 1).

### Intrahepatic

Fibroblast growth factor receptor (FGFR) is one of the important targets in CCA targeted therapy. FGFR protein mainly includes four subtypes: FGFR1, FGFR2, FGFR3 and FGFR4, all of which have the structural characteristics of the extracellular domain, transmembrane domain, and receptor phosphorylated intracellular domain that bind to ligands. They are tyrosine kinase signaling pathways, and part of it is involved in cell proliferation and differentiation. FGFR1 is mainly distributed in breast and lung cancer, FGFR2 is mainly distributed in liver cancer, and FGFR3 is mainly distributed in urothelial cancer 50. In ICCA, the main manifestation is FGFR2 gene fusion 51. A study from Japan showed that 4.8% of perihilar cholangiocarcinoma also carry FGFR2 fusion, while the frequency of FGFR2 fusion in ICCA is 7.7% 52, which may be related to hilar and intrahepatic are not related to a clear anatomical classification, but this also shows the complexity of FGFR2 fusion mutations in CCA. The FIGHT-202 study uses FGFR1, FGFR2 and FGFR3 selective competitive inhibitor Pemigatinib to treat patients with locally advanced or metastatic CCA with FGFR2 fusion or rearrangement. The treatment response rate is as high as 35.5%, making Pemigatinib hopeful to become FGFR2 fusion or new treatment for rearrangement of advanced CCA 53.

Isocitrate dehydrogenase (IDH) participates in the citric acid cycle, IDH 1/2 gene mutations can cause intracellular DNA methylation and hypoxia, and promote tumor formation. Approximately 14% of ICCA patients carry IDH gene abnormalities, which occur less frequently in ECCA patients, and IDH 1 gene abnormalities are more common than IDH 2. Phase III clinical trials of IDH 1 inhibitors have been shown to have a clear disease control effect on patients with CCA 54. Ivosidenib compared with placebo to treat advanced CCA with IDH 1 mutations that failed first-line chemotherapy. The results of the Phase III clinical trial showed that the experimental group had progression-free survival (PFS) 2. 7 months, prolonged the OS to 10.8 months, the risk of disease progression or death was reduced by 63%, and the effect was significantly better than that of the placebo group.

The expression of vascular endothelial growth factor receptor (VEGF) is found in 30%-40% of CCA patients, and is associated with LNM and poor prognosis. The BRAF gene mutation is basically not present in ECCA, but it exists in ICCA a 3.3% of mutation 55.

### Extrahepatic

The ERBB receptor tyrosine kinase family includes 4 cell surface receptors: ERBB1/EGFR, ERBB2/HER-2, ERBB3 and ERBB4, which play a pivotal role in the process of cell differentiation and regulation 56. HER-2 is mainly found in ECCA, Nam 57 found that through preclinical experiments on CCA cells and mouse models, the drug named trastuzumab for this target showed a good anti-tumor effect, indicating that HER-2 may be a potential target of HER-2 gene inhibitors is slightly better in patients with gallbladder cancer. There is mainly preclinical data instead of few reports in patients with CCA, and corresponding clinical drug research is temporarily lacking.

Epidermal growth factor receptor (EGFR) also has a higher mutation rate in ECCA than ICCA. Nowadays, the current progress in clinical research on EGFR has not achieved significant benefits, a Meta-analysis evaluated the addition of EGFR monoclonal antibody to Gemcitabine-based first-line chemotherapy in advanced CCA. The results showed that there was no statistical significance in OS, PFS and ORR 58, and the combination therapy significantly increased blood and skin adverse effects reaction. The difference is that the local administration of Gemcitabine reported by Korean scholars can promote the degradation of EGFR and inhibit the growth of CCA 59. The synergistic mode of Gemcitabine and EGFR have brought new inspiration to future research on the treatment of CCA.

Mitogen-activated extracellular signal-regulated kinase (MEK) is a key substance in the cell signaling pathway and participates in the growth of tumor cells. Compared with ICCA, ECCA has a higher expression 60. Binimetinib, as a potent and highly selective MEK1/2 inhibitor, combined with Capecitabine in the second and third-line treatment of CCA patients who have failed first-line chemotherapy has a DCR as high as 76.5%, ORR is 20.6% 61. These encouraging clinical research results provide strong evidence for the further exploration of MEK inhibitors in CCA.

For targeted therapy related to signaling pathways, the PI3K/Akt/mTOR signaling pathway plays an important role. This pathway is not only involved in regulating the proliferation, differentiation and apoptosis of CCA cells, but also has a regulatory role in the function of CCA microenvironmental cells. Mutation of PIK3CA, the coding gene of PI3K, increases the activity of PI3K, a kinase in CCA cells, which not only reduces apoptosis of cancer cells, but also improves the infiltrating growth ability of CCA. Activated PI3K can cause Akt phosphorylation, and p-Akt regulates the cycle and apoptosis of cancer cells by affecting the expression of caspase-9 and nuclear factor-κ B, which play an important role in the growth and invasion of CCA 62. The Ras/Raf/MAPK signaling pathway is one of the main pathogenic mechanisms of CCA. It is regulated by a variety of receptor molecules such as EGFR, HER2, VEGFR, and can interact with the cyclin p21 and PI3K/Akt signaling pathways. And it regulates the proliferation, differentiation and survival of tumor cells. Moreover, in the early stage of CCA, after the mutation of the K-Ras gene, the molecular switch of this pathway in the bile duct epithelial cells, the normal Ras protein cannot be produced, which leads to disorder of the signal pathway, uncontrolled cell proliferation and cancer 63. The Notch signaling pathway, which is highly conservative in evolution, participates in the regulation of a variety of malignant biological behaviors of tumor cells, and plays an important role in the process of CCA biogenesis. Liu et al. 64 found that the expression of miR-129-2-3p in ICCA tissues and cells was reduced, and its target gene Wip1 played a tumor-suppressing effect in the progression of ICCA. The high level of Wip1 in human ICCA is associated with metastasis to lymph nodes (P=0.022). Compared with the control group, Wip1 gene deletion can significantly inhibit the proliferation and invasion of ICCA cells. Therefore, Wip1 may be a key regulator of human ICCA tumorigenesis and invasion, and Wip1 may be a therapeutic target of ICCA 65. At the same time, the low expression of iNOS can also inhibit the proliferation of ICCA cells and promote cell apoptosis 66, which can be used as a breakthrough in the treatment of CCA in the future.

In recent decades, targeted therapy studies for CCA have shown no clear effect. On the one hand, the common target mutation rate is very low, and the treatment effect is poor. On the other hand, in addition to common targets, there are many unknown targets and we have not found a way to deal with them. Due to the development of next-generation molecular sequencing technology and the discovery of some emerging targets, the bile duct cancer gene map has been further improved, and as a novel treatment method for CCA, targeted therapy has a broad field of exploration, and it is worthwhile to spend more energy on research. In the future, targeted therapy is likely to become a boon for patients with CCA.

## Immunotherapy

### Immune checkpoint inhibitors

Immune checkpoint inhibitors are the focus of immunotherapy in recent years. Their anti-tumor effects are mainly through blocking immune detection sites, re-establishing normal anti-tumor immunity, restoring or improving the body's anti-tumor immune response, and thus controlling and clearing tumors. Immune checkpoints are molecules that regulate the immune system's response to foreign invaders. Under normal conditions, co-stimulatory molecules are balanced with immune checkpoint molecules to minimize the invasion of surrounding normal tissues 67. Immune checkpoints are often manipulated by tumor cells to evade immune surveillance, such as Programmed death protein-1 (PD-1) and Cytotoxic T-lymphocyte antigen-4 (CTL-4). These checkpoints, once activated by their specific ligands (PD-L1 and CD152), respectively, promote apoptosis in peripheral blood T cells 68. In recent years, checkpoint inhibitors such as CTLA-4 and PD-1 have been found to enhance the antitumor immune response 69.

PD-1 has two binding ligands, PD-L1 and PD-L2, of which PD-L1 is the most prominent in regulation. PD-1 binds to PDL-1 and PDL-2 to inhibit the immune activity of T cells and promote tumor growth. Blocking PD-1 can restore T cell immune activity, thereby improving anti-tumor activity. At present, PD-1/PD-L1 inhibitors mainly include Nivolumab, Pembrolizumab, Avelumab, Durvalumab, Atezolizuma and many more, and they have achieved good curative effects in the treatment of non-small cell lung cancer, malignant melanoma, and renal cell carcinoma 70, and have been found effective for malignant tumors such as stomach cancer and liver cancer 71. In 2015, the European Society of Medical Oncology (ESMO) reported the interim results of the KEYNOTE-028 phase Ib study. 24 patients with advanced biliary tumors (20 cases of CCA, 4 cases of gallbladder) with positive PD-L1 expression (>1%), the objective remission rate of the anti-PD-1 monoclonal antibody Pembrolizumab treatment was 17%, 4 cases had partial remission, 4 cases had stable disease, the drug showed good antitumor activity, and the patients were well tolerated. Alshari OM et al. 72 reported a case of Pembrolizumab applied to CCA patients, and there was no recurrence after 2 years of follow-up. However, there is still a lack of data on the effectiveness and safety of the drug in large-scale use of CCA. In addition, there are many other different combinations of Pembrolizumab in clinical research on advanced CCA. For example, ASCO reported in 2018 the results of a phase II clinical trial of Pembrolizumab combined with granulocyte-macrophage colony stimulating factor in the treatment of advanced biliary tract tumors. Of the 27 patients (74% ICCA), 5 cases (19%, 1 case of microsatellite instability, 4 cases of microsatellite stable) achieved partial remission, and the 6-month disease-free survival rate reached 35%. 30% of the cases are PD-L1 positive (≥1%), but there is no correlation with the objective response rate and the 6-month disease-free survival rate. Mou H et al. 73 reported a case of ICCA with high tumor mutational burden (TMB) and PD-L1 expression after receiving Pembrolizumab treatment combined with complete remission (CR) with fewer side effects. It seems that both PD-L1 expression and TMB may be potential indicators for predicting treatment response 74. The expression of PD-L1 in tumor cells, tumor mutation burden, high degree of microsatellite instability-high (MSI-H)/mismatch repair deficiency (dMMR), and many more can often be used to assess the sensitivity of immune checkpoint inhibitors of biomarkers. In 2017, Le et al. 75 reported the efficacy of Pembrolizumab in dMMR solid tumors. The study included 86 patients with 12 tumor types, with an objective response rate of 53%. Including 4 cases of CCA, 1 case had complete remission, 3 cases had stable disease, and the disease control rate was 100%. It has been previously reported that 5%-10% of CCA patients have dMMR 76, and 321 cases of biliary tract tumors have been sequenced. 13% of ICCA patients, 26% of ECCA patients, and 6% of gallbladder cancers have DNA repair mutations (including MSH6, BRCA1, BRCA2, ATM, MLH1, MSH2 and many more). In dMMR cancers, the proportion of mutated neoantigens is large, which makes them sensitive to immune checkpoints, regardless of the origin of the cancer 77. The above conclusions indicate that checkpoint inhibitors may be a promising treatment for dMMR/MSI-H CCA patients. Nivolumab is an IgG4 monoclonal antibody that has human immune function and targets PD-1. Gou et al. 78 used Nivolumab to treat 30 patients with metastatic BTC. The objective response rate (ORR) was 20%, the disease control rate (DCR) was 60%, and the PFS was 3.1 months without serious adverse reactions. This also shows that Pembrolizumab and Nivolumab have potential curative effects for the advanced treatment of CCA.

CTLA-4 is a member of the immunoglobulin superfamily and is mainly located in the cells of quiescent primitive T cells. CTLA-4 interferes with the binding of B7 and CD2 by directly transmitting inhibitory signals to T cells, thereby inhibiting the response of T cells 79. The blocking agent not only increases the number of CD8+ T cells, but also consumes Treg cells in the tumor to improve the body's immune activity 80. CTLA-4 inhibitors mainly include Tremelimumab and Ipilimumab. Currently, the evaluation of the efficacy and safety of CTLA-4 inhibitors related to CCA is still in clinical trials. The Xie 81 used Tremelimumab combined with microwave ablation to treat refractory biliary tumors. The median PFS and OS of patients were 3.4 months and 6.0 months, respectively, suggesting the potential of Tremelimumab in the treatment of advanced gallbladder cancer.

With the development of relevant clinical trials in recent years, more and more clinical results have proved that the efficacy of immune checkpoint inhibitors is significant. Therefore, it is a good choice to add immune checkpoint inhibitors to the treatment of CCA.

### Tumor vaccine

Tumor vaccines can activate the patient's own immune system, enhance immunogenicity, and induce specific cellular and humoral immune responses in the body using tumor cells or protoplasmic substances to stop tumor growth, infiltration and recurrence, resulting in tumor clearance or control. There are two cancer-related antigens used in vaccine therapy, namely Wilms' tumor gene (WT1) antigen and mucin 1 (MUC1) 68, which is mainly a DNA-binding transcription factor and is present in 80% of biliary tract tumors. MUC-1 is an epithelial glycoprotein and is overexpressed in 90% of biliary tract tumors. Currently, there are mainly single-antigen vaccines, multi-antigen vaccines and dendritic cell vaccines, and the latter two are the main research directions of tumor vaccines.

Single-antigen vaccine: This single-peptide based vaccine therapy is usually well tolerated immunologically but exerts a more limited antitumor effect 82. In a clinical trial of WT1 vaccine combined with Gemcitabine for advanced biliary tract and pancreatic cancer, enrolling 25 patients, 8 of whom had CCA, the combination therapy was associated with adverse events comparable to Gemcitabine alone. Cell culture experiment showed the presence of WT1-specific T cells in 59% of patients, a delayed hypersensitivity response after vaccination in 2 patients, a median OS of 288 days in CCA patients, and a 2-month DCR of 89% 83. The clinical efficacy and adverse effects, although not significantly different, demonstrated the safety of the combination therapy of WT1 vaccine and GEM.

#### Multi-antigen vaccine

The effect of multiple tumor antigen peptide mixed vaccine is better than single peptide vaccine, and because long peptides can be recognized by MHC-Ⅰ and MHC-Ⅱ molecules at the same time, it can induce CD8+ T cell response and CD4+ T cell response. It can be seen that long peptides have more advantages than short peptides. The use of individualized peptide vaccines in combination with low-dose cyclophosphamide in patients can significantly prolong the progression-free survival and overall survival of patients compared with the use of vaccines alone. The difference is statistically significant (6.1 months vs 2.9 months, P = 0.008; 12.1 months vs 5.9 months, P=0.004) 84. Aruga et al.^85^ reported a phase I clinical trial of peptide vaccination in patients with advanced BTC, the trial selected 9 patients with advanced BTC and received subcutaneous injections of 3 HLA-A*2402 restricted epitope peptides-cell division associated 1 (CDCA1), cadherin 3 (CDH3) and kinesin family member 20A (KIF20A). Peptide-specific T cell immune responses were observed in all patients. In 9 patients, 5 patients were in stable disease (SD), and the median PFS and OS of the patients were 3.4 months and 9.7 months, respectively. It can be seen that peptide vaccines overcome the limitations of single peptide vaccines by immunizing patients against multiple antigens, thereby increasing the response rate of tumor cells to antigens 86.

#### Dendritic vaccine

Dendritic cells (DC) are the most powerful and effective antigen-presenting cells, which induce the body to produce cytotoxic T cells and mediate specific anti-tumor cell immunity 87. Similar to peptide vaccines, dendritic cell vaccines expose the immune system to antigens with the purpose of generating memory lymphocytes, thereby generating a powerful secondary immune response 88. In a retrospective study 89, 65 patients with unresectable, recurring, or metastatic CCA were selected to receive DC-based immunotherapy, and WT1 and/or MUC1 pulses were used to stimulate DC. The results showed that the regimen was well tolerated, and 15% of patients were in stable condition after 6 months of treatment. A recent study found that Honokiol can enhance the immunogenicity of BTC cells, thereby increasing the effectiveness of DC vaccines 90. The above research shows that DC vaccine combined with chemotherapy can prolong the survival time of patients more than DC vaccine alone.

The effect of tumor vaccines on patients with CCA needs further research and verification. It is believed that tumor vaccines can become a consideration for adjuvant treatment of advanced CCA.

### Adoptive Cell Transfer therapy

Adoptive cellular immunotherapy is the separation of autologous or foreign immune cells, and after *in vitro* activation or genetic modification, a sufficient amount of anti-tumor active immune cells is amplified and transferred back to tumor patients to amplify the cellular immune function in the patient's body and relieve Tumor immune tolerance to improve the anti-tumor effect of the treatment method 91. There is a case report of a 30-year-old male with stage IV CCA with mediastinal LNM. After liver transplantation, the patient received allogeneic γδ T cell immunotherapy because of recurrence of CCA with mediastinal LNM. After that, the size and activity of the lymph nodes were significantly reduced, the immune function was improved, and no complications related to γδ T cell infusion were observed. This case shows that exogenous γδ T cell immunotherapy can be developed into a promising new method for the treatment of CCA 92. A patient with metastatic CCA who progressed after multi-line chemotherapy participated in a phase II clinical trial of adoptive cell therapy. The trial first reinfused CD4 + ErbB2IP mutation-specific T cells, and then applied cytokine IL-2 to enhance T cell proliferation. After 7 months, all tumor metastases shrank by 30%, and tumors reached partial remission. After 13 months of stable disease, only lung metastases progressed slightly, and liver metastases were stable 93, suggesting the use of auto-expanded T cells targeting specific tumor antigens in the patient's BTC feasibility.

Immunotherapy is an anti-tumor method that has gradually received attention in recent years. Due to the complexity of the tumor microenvironment of CCA, in addition to mesenchymal and endothelial cells, a large number of immune cells also play an important role. At the same time, there is also a natural and adaptive immune system. In the future, the research prospects of immunotherapy are also very broad and of great significance. This research will play a key role in the treatment of CCA, and will be more conducive to improving the survival rate of patients and improving the prognosis.

## TACE

Transcatheter arterial chemoembolization (TACE) is one of the first-line treatment options for patients with unresectable ICCA. It uses prognostic factors to hierarchically screen patients with ICCA who are sensitive to TACE treatment, and can provide individualized treatment for them to improve the treatment effect and survival benefits of patients. In clinical studies such as Li 94, 211 patients with ICCA who underwent R0 resection were included (68 patients underwent adjuvant TACE after operation and 143 patients without adjuvant TACE). Multivariate analysis found that not receiving postoperative adjuvant TACE treatment was a high-risk factor affecting postoperative survival (HR=1.77, 95% confidence interval 1.15 to 2.73, P = 0.010). In the multivariate analysis for postoperative recurrence, not receiving postoperative adjuvant TACE can reduce postoperative recurrence (HR=0.59, 95% confidence interval: 0.38 to 0.92, P = 0.020). At the same time, the recurrence rate of patients receiving TACE treatment in TNM Ⅰ phase is higher than that of patients without TACE treatment (18/35 vs. 15/63, P = 0.006). Research suggests that TACE can promote the formation of local vascular growth factors. Thereby promoting tumor metastasis, especially in patients with TNM stage I. For patients with TNM Ⅱ to Ⅲ stage, TACE can prolong the OS of the patient (P = 0.020), but it cannot reduce postoperative recurrence. Therefore, the study believes that TACE therapy may be beneficial for patients with late-stage tumors. A recent study from Ruijin Hospital on postoperative adjuvant TACE treatment of hepatitis B-related ICCA included a total of 9 postoperative adjuvant TACE cases and 33 cases of only manual treatment 95. There are significant differences in 1, 3, and 5-year survival rates between the postoperative TACE group and the TACE-free group, which are 88.9%, 77.8%, 66.7% vs. 63. 6%, 30.8%, 13% (P = 0.037). At the same time, multivariate analysis also confirmed that postoperative adjuvant TACE can improve the long-term survival of patients after surgery (HR: 0.123, 95% CI: 0.023 to 0.643, P = 0.013).

In clinical practice, TACE has been used to treat ICCA 95, but it has limitations when used alone, it can be treated together with percutaneous thermal ablation. The possibility of percutaneous thermal ablation to achieve a complete ablation decreases as the size of the tumor increases. In the clinical study of WU et al., 53 patients were treated with a total of 83 times of percutaneous thermal ablation and simultaneous TACE treatment. The effective rate was 80.7%, and the median PFS and median OS were 7.2 months and 20.9 months. The 1, 2, and 3-year cumulative survival rates were 72.6%, 39.1%, and 24.3%. The cumulative PFS rates at 6, 12, and 18 months were 58.3%, 40.4%, 24.2%. This study shows that percutaneous thermal ablation with simultaneous TACE treatment for advanced ICCA is safe and effective.

Nowadays, TACE is a mature method for the treatment of liver and biliary tumors. The benefits of patients are also obvious to all, but the adverse reactions brought about by it need to be paid attention to in the treatment.

## Others

Radio frequency ablation (RFA) uses high-frequency alternating current to release heat energy through electrode needles, which in turn leads to local tissue necrosis. RFA has become a recognized therapy for CCA due to its efficacy, safety and availability 96. Meta-analysis showed 97 that patients who were treated with RFA before biliary stent implantation, compared with patients who did not receive RFA, the stent patency time and survival time were prolonged by 13 d and 37 d, respectively, highlighting the treatment of RFA combined with malignant biliary obstruction advantage. Percutaneous transhepatic puncture of biliary tract cavity RFA combined with biliary stent implantation is a safe and feasible treatment method for the treatment of malignant distal biliary obstruction 98. Endoscopic bile duct radiofrequency ablation (EB-RFA) is also a new way to treat CCA. Kim et al. 99 conducted a retrospective study on 8 DCCA patients who received EB-RFA before surgery, and the results showed that the technique was safe and effective without serious complications. A previous report showed that survival was significantly better in patients with stage I ICCA (single tumor, no vascular invasion) who underwent radiofrequency ablation compared with no treatment 100. However, percutaneous ablation therapy was performed as initial treatment for ICCA in only 3.6% according to the Report of the 20^th^ Japanese national follow-up survey. In the SEER database from the United States, only 5.2% of ICCA patients received percutaneous ablation therapy alone. Therefore, percutaneous ablation therapy is limited to clinical practice. However, major limits are high local recurrence frequencies, mainly for lesions larger than 3 cm, and a potential incomplete ablation of lesion near large vessels (>3 mm in diameter) 101, 102.

Photodynamic therapy (PDT) is a kind of ablation therapy. The principle is that the photosensitizer injected into the body can specifically accumulate in malignant tumor cells. After these specifically labeled tumor cells are irradiated with a specific wavelength of laser light, a series of toxic substances such as singlet oxygen and free radicals are generated through photochemical reactions, thereby killing tumor cells. On the basis of PDT and supplemented drug treatment, patients can often avoid the pain of traditional surgery while obtaining good treatment results, thereby improving survival time. It has been used in the palliative treatment of CCA for extrahepatic patients. PDT has the advantages of wide applicability, low side effects, low damage, simple and easy operation, and low postoperative recurrence rate. Wentrup R et al. 103 and Park DH et al. 104 respectively compared and studied the efficacy of PDT combined with Gemcitabine, PDT combined with TGO and PDT alone. Both studies have proved that PDT combined with chemotherapy can enhance efficacy, prolong patient survival, and reduce mortality. It can be seen that the application of PDT in patients with unresectable ECCA can alleviate the clinical symptoms of patients, reduce tumor volume, delay tumor growth, improve quality of life, and prolong the survival of patients.

Compared with traditional surgery, microwave ablation (MWA) has many theoretical advantages, including less dependence on tissue conductivity, short ablation time, high intratumoral temperature, and a larger and uniform ablation zone 105. There were no surgery-related deaths in ICCA patients treated with MWA, which was significantly lower than the reported perioperative mortality (1.2% ~ 7%). The incidence of major complications related to surgery is significantly lower than that after surgery, reportedly ranging from 11% to 58%. The survival time of ICCA patients is significantly improved compared with pure palliative treatment, and the survival rate after radical resection is equivalent 106. These results suggest that MWA is less traumatic than surgical treatment and is safe and effective for ICCA.

## Conclusion

**Figure 2:**
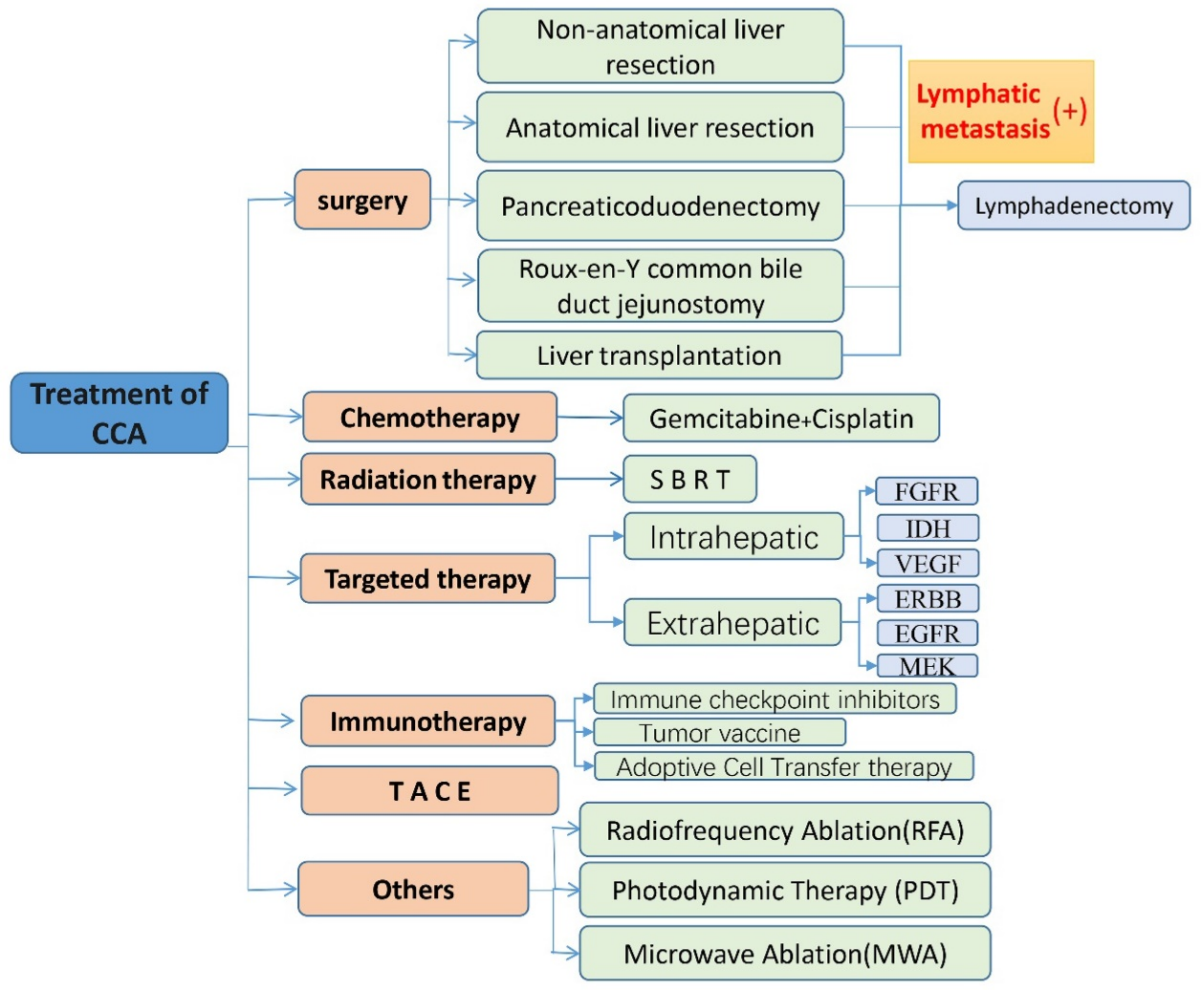
Treatments for cholangiocarcinoma.

**Figure 3:**
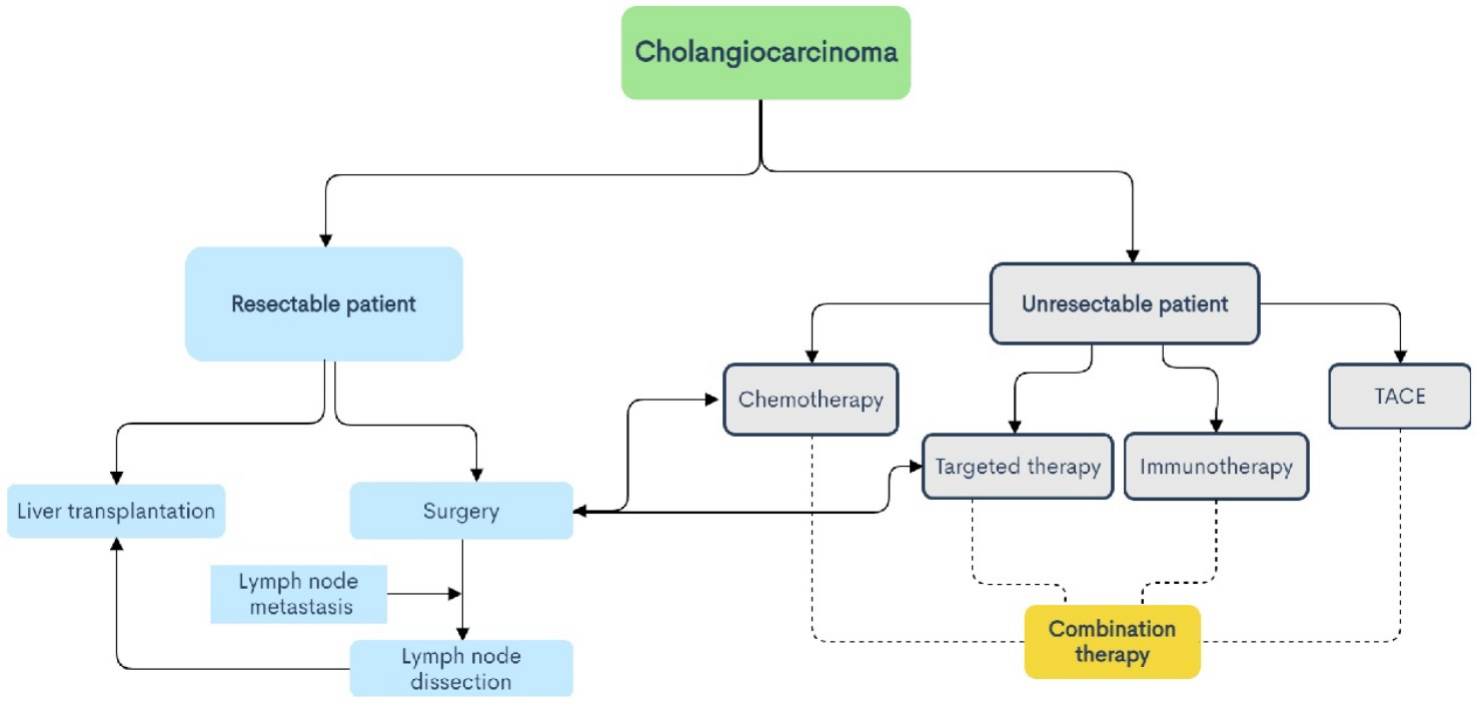
Treatment planning process for cholangiocarcinoma.

CCA is a type of tumor with high molecular diversity and genetic heterogeneity, and has a high incidence in Asia. With the increase in the incidence of CCA year by year, people's understanding and attention to it are increasing, but the ideal treatment is still surgical treatment. For advanced patients and patients with high-risk recurrence factors, high invasiveness, positive resection margins, or LNM, radiotherapy, chemotherapy, targeted therapy, immunotherapy, TACE and other treatment methods can benefit some patients (shown in Fig. 2). The combination of Gemcitabine and Cisplatin is still the current first-line chemotherapy regimen for patients with advanced CCA, and the combination of paclitaxel and other drugs with Gemcitabine is worth exploring. Radiotherapy is also considered to be a type of locally advanced unresectable CCA. Effective local treatment methods are gradually being used clinically to verify his effects. With the development of next-generation gene sequencing technology and the discovery of some emerging targets, targeted therapy has gradually become a hot spot in the treatment of CCA. Different signal pathways are involved in many biological behaviors of tumor cells, so they play an important role in the diagnosis and treatment of CCA. Immunotherapy such as immune checkpoint inhibitors has also been the focus of treatment for CCA in recent years, and tumor vaccines also have certain benefits. TACE can provide patients with personalized treatment and improve the treatment effect, and its combination with other treatment options is also proven to be safe and effective. Nowadays, some ablation techniques such as radiofrequency ablation and photodynamic therapy are gradually confirming that they have certain effects in the treatment of CCA, inhibiting tumor growth, delaying the progression of the disease, and improving the prognosis of patients. In addition, advanced and unresectable CCA is insensitive to radiotherapy and chemotherapy due to heterogeneity and interstitial components. In short, targeted therapy and immunotherapy have many unknown areas that we need to explore, and they are also the focus of our attention. The exploration of immune checkpoint inhibitors in the treatment of CCA is in the ascendant. The current second-line treatment for advanced biliary cancer is the combination of immune checkpoint inhibitors and molecular targeted drugs, the combination of immune checkpoint inhibitors, and the combination of immune checkpoint inhibitors and other treatments, the combined application is also being studied in depth, and the results are worth looking forward to. Perhaps this is the future hope for the treatment of CCA. However, the existing diagnostic methods are still lacking in the early diagnosis of CCA, and because of the differences in molecular pathology and gene mutations between tumors at different anatomical sites, and the effects of different treatment plans are also quite different (shown in Fig. 3). Understanding the subtypes and mutant genes of CCA is of great significance for the precise treatment of CCA. In the future, with the development of multiple disciplines such as genetic engineering, molecular biology, and tumor immunology, more effective biomarkers will be discovered, and clinical trials that actively investigate immunotherapy and the efficacy of combination with radiotherapy and chemotherapy, targeted therapy and many more. Development is of great significance for the establishment of a new and effective treatment model for patients with CCA.

## Figures and Tables

**Table 1:** Clinical Trials of Chemotherapy, Targeted Therapy and Immunotherapy for Cholangiocarcinoma

	Trial description	Pathways	Relevant data	(Median) overall survival	Clinical Trials.gov reference
**Chemotherapy**					
FOLFOX	phase III trial	Chemotherapy	DCR=33%	6.2 month	NCT01926236
FOLFIRINOX	phase II trial	Chemotherapy	ORR=10%	10.7 month	NCT02456714
			PFS=6.2 month		
**Targeted Therapy**					
Derazantinib	phase II trial	FGFR	ORR=20.7%	13.4 month (lower expected)	NCT03230318
			DCR=82.8%		
Pemigatinib	phase II trial	FGFR	ORR=35.5%	21.2 month	NCT02924376
Erdafitinib	phase II trial	FGFR	CRR=3%	13.8 month	NCT02365597
			PRR=37%		
Ivosidenib	phase II trial	IDH1	PFS(I)=61%	10.8 month	NCT02989857
			PFS(F)=82%		
Ramucirumab	phase II trial	VEGF	—	5.2 month	NCT01170663
Trastuzumab	phase II trial	HER2	HR=0.76	—	NCT00045032
			DEAD=0.74		
Niraparib	phase II trial	MAPK	DEAD=16.1%	21.0 month	NCT01847274
**Immunotherapy**					
Durvalumab (Anti‑PD‑L1 antibody)	phase I trial	PD-L1	HR=0.73	13.0 month	NCT03043872
			AE=5%		
Pembrolizumab	phase II trial	PD-1	ORR=34.3%	23.5 month	NCT02628067
Nivolumab (ti‑PD‑1 antibody)	phase II trial	PD-1	ORR=22%	14.2 month	NCT02829918
			DCR=59%		
